# Supplementary material for: Sperm Quality Affected by Naturally Occurring Chemical Elements in Bull Seminal Plasma
Source: Antioxidants (Basel). 2022 Sep 12;11(9):1796. doi: 10.3390/antiox11091796 (PMC9495912; doi:10.3390/antiox11091796)
Supplement: Supplementary file 1 [file antioxidants-11-01796-s001.zip › antioxidants-1834609-supplementary.pdf]

Supplementary Materials

Table S1. Correlations: spermatozoa parameters vs markers of OS. biochemical parameters. and chemical elements.

		Al	Ba	Ca	Cd	Co	Cr	Cu	Fe	Hg	K	Mg	Mn	Mo	Na	Ni	Pb	Sr	Zn
TOS	r	-0.12	-0.12	0.08	-0.09	-0.03	-0.13	0.63	-0.04	-0.32	-0.02	0.52	0.11	-0.26	-0.04	-0.24	-0.12	-0.19	-0.11
	p	0.658	0.659	0.769	0.750	0.917	0.633	**0.008**	0.897	0.309	0.935	**0.039**	0.681	0.336	0.883	0.366	0.667	0.479	0.684
FRAP	r	-0.18	-0.18	0.08	-0.25	-0.12	-0.17	-0.14	0.02	0.26	-0.15	-0.21	0.00	-0.09	0.20	-0.05	-0.16	-0.21	0.33
	p	0.496	0.480	0.765	0.334	0.651	0.509	0.590	0.950	0.385	0.574	0.419	0.993	0.737	0.431	0.840	0.527	0.410	0.202
SOD	r	-0.20	-0.19	0.09	-0.22	-0.14	-0.18	0.13	0.01	0.33	-0.31	0.28	-0.02	-0.36	0.33	-0.08	-0.18	-0.25	-0.03
	p	0.441	0.461	0.732	0.393	0.593	0.484	0.613	0.972	0.276	0.222	0.280	0.942	0.154	0.202	0.761	0.500	0.333	0.901
GPx	r	-0.42	-0.39	-0.25	-0.36	-0.45	-0.41	-0.24	-0.53	-0.61	-0.27	-0.21	-0.31	-0.28	0.10	-0.08	-0.42	-0.39	-0.13
	p	0.093	0.125	0.328	0.151	0.072	0.100	0.355	**0.028**	**0.027**	0.297	0.419	0.231	0.270	0.698	0.764	0.096	0.120	0.610
MDA	r	0.05	0.10	0.20	-0.03	0.10	0.06	0.14	-0.02	0.35	-0.33	-0.04	-0.42	0.20	0.21	0.37	0.06	0.16	-0.16
	p	0.861	0.716	0.458	0.915	0.701	0.828	0.600	0.949	0.272	0.208	0.893	0.109	0.467	0.434	0.156	0.837	0.566	0.559

Numbers in bold represent the significant correlations.

Table S2. Correlations: markers of OS vs chemical elements.

		TOS	FRAP	SOD	GPx	MDA	TP	Alb	Chol	TAG	Al	Ba	Ca	Cd	Co	Cu	Fe	Hg	K	Mg	Mn	Mo	Na	Ni	Pb	Sr	Zn
Concentration	r	0.18	-0.04	-0.14	0.16	0.02	-0.03	0.03	0.12	0.21	0.05	0.04	-0.11	0.08	0.08	0.05	-0.17	0.02	-0.49	-0.29	-0.31	0.17	-0.02	0.18	0.09	0.03	0.03
	p	0.505	0.873	0.590	0.546	0.939	0.897	0.904	0.627	0.422	0.845	0.877	0.678	0.751	0.740	0.856	0.491	0.932	0.074	0.248	0.205	0.502	0.923	0.484	0.719	0.896	0.905
MOT_0	r	0.12	0.28	0.28	-0.21	0.27	-0.30	-0.11	-0.59	-0.24	0.20	0.16	0.25	0.12	0.25	0.19	0.10	-0.26	0.21	-0.32	0.03	0.10	0.02	0.32	-0.06	0.19	0.13
	p	0.656	0.279	0.280	0.424	0.304	0.246	0.671	**0.009**	0.351	0.424	0.527	0.319	0.641	0.309	0.454	0.688	0.305	0.481	0.192	0.900	0.706	0.943	0.192	0.823	0.462	0.621
MOT_1	r	0.08	0.02	0.10	-0.34	0.05	-0.07	0.25	-0.31	0.03	-0.41	-0.43	0.33	-0.42	-0.32	-0.41	0.27	0.48	0.29	-0.09	0.09	-0.37	-0.14	0.27	0.15	-0.42	-0.44
	p	0.781	0.930	0.689	0.181	0.840	0.776	0.330	0.214	0.898	0.092	0.073	0.184	0.080	0.188	0.087	**0.043**	0.275	0.323	0.731	0.717	0.129	0.585	0.287	0.564	0.083	0.071
PRO_0	r	0.16	0.22	0.26	-0.17	0.30	-0.28	-0.11	-0.50	-0.21	0.24	0.21	0.16	0.17	0.30	0.23	0.09	-0.30	0.15	-0.39	0.05	0.15	-0.03	0.37	-0.12	0.23	0.18
	p	0.543	0.399	0.322	0.519	0.265	0.268	0.687	**0.033**	0.428	0.328	0.401	0.524	0.506	0.235	0.348	0.734	0.224	0.604	0.107	0.831	0.540	0.913	0.129	0.637	0.355	0.476
PRO_1	r	0.10	0.05	0.12	-0.31	0.09	-0.11	0.23	-0.30	0.05	-0.43	-0.45	0.33	-0.44	-0.34	-0.43	0.27	0.47	0.29	-0.10	0.07	-0.39	-0.15	0.29	0.14	-0.44	-0.45
	p	0.723	0.848	0.634	0.225	0.733	0.687	0.373	0.223	0.845	0.076	0.062	0.188	0.065	0.168	0.073	**0.046**	0.274	0.316	0.679	0.771	0.106	0.564	0.236	0.567	0.070	0.062
VCL_0	r	0.04	-0.25	0.22	-0.26	0.08	0.07	0.20	-0.03	-0.18	0.19	0.15	0.12	0.25	0.15	0.19	0.00	-0.19	0.48	-0.12	0.53	0.19	-0.27	0.09	-0.22	0.19	0.16
	p	0.869	0.337	0.390	0.321	0.780	0.783	0.449	0.902	0.479	0.458	0.547	0.637	0.325	0.555	0.455	0.985	0.460	0.081	**0.024**	0.622	0.438	0.274	0.721	0.388	0.443	**0.049**
VCL_1	r	0.40	0.16	0.47	0.10	0.40	-0.31	-0.05	-0.02	0.15	0.18	0.17	0.11	0.17	0.18	0.18	0.03	-0.29	0.36	-0.03	0.21	0.16	-0.26	0.05	-0.34	0.19	0.20
	p	0.123	0.535	0.056	0.698	0.124	0.221	0.859	0.944	0.578	0.465	0.503	0.662	0.489	0.467	0.485	0.917	0.239	0.209	0.899	0.406	0.521	0.306	0.858	0.163	0.439	**0.043**
BCF_0	r	0.09	-0.14	-0.14	0.20	0.16	0.11	0.07	0.35	0.34	0.04	0.06	-0.13	0.05	0.02	0.04	-0.02	0.06	0.14	0.11	-0.06	0.04	-0.08	-0.15	-0.15	0.05	0.12
	p	0.732	0.591	0.605	0.440	0.546	0.683	0.791	0.151	0.184	0.888	0.813	0.611	0.843	0.952	0.887	0.939	0.801	0.632	0.661	0.822	0.873	0.744	0.555	0.556	0.857	0.648
BCF_1	r	0.11	-0.06	-0.05	0.47	0.41	0.04	-0.02	0.47	0.11	0.03	0.09	-0.41	0.03	0.02	0.04	-0.18	-0.36	-0.33	-0.15	0.02	-0.03	-0.05	-0.02	-0.20	0.05	0.14
	p	0.689	0.811	0.860	0.057	0.118	0.876	0.954	**0.048**	0.675	0.903	0.717	0.088	0.908	0.943	0.863	0.487	0.147	0.244	0.561	0.941	0.907	0.846	0.932	0.423	0.830	0.589
ALH_0	r	0.09	0.05	0.28	-0.20	0.01	-0.13	0.04	-0.36	-0.31	0.17	0.13	0.26	0.20	0.18	0.17	0.06	-0.27	0.17	-0.16	0.37	0.16	-0.09	0.14	-0.05	0.17	0.09
	p	0.727	0.861	0.274	0.435	0.958	0.620	0.866	0.141	0.227	0.490	0.610	0.303	0.429	0.465	0.498	0.823	0.287	0.571	0.525	0.133	0.516	0.708	0.569	0.856	0.508	0.733
ALH_1	r	0.18	0.04	0.24	-0.26	-0.12	-0.12	0.03	-0.34	0.03	0.03	-0.02	0.48	0.05	0.07	0.02	0.23	0.12	0.49	0.19	0.23	0.11	-0.11	0.00	-0.04	0.03	-0.05
	p	0.497	0.888	0.361	0.322	0.664	0.639	0.907	0.167	0.900	0.896	0.942	**0.042**	0.854	0.784	0.945	0.355	0.622	0.076	0.443	0.359	0.660	0.655	0.997	0.881	0.921	0.853
MTT	r	0.21	-0.02	0.26	-0.18	0.44	-0.13	0.11	-0.05	-0.20	-0.04	-0.03	-0.07	-0.03	0.04	-0.04	0.22	0.25	0.12	-0.01	0.53	-0.06	0.01	-0.13	0.20	-0.04	-0.03
	p	0.461	0.950	0.357	0.530	0.119	0.656	0.704	0.854	0.467	0.870	0.898	0.807	0.911	0.881	0.893	0.407	0.348	0.708	**0.034**	0.975	0.827	0.976	0.638	0.457	0.878	0.912

Numbers in bold represent the significant correlations.
